# Supplementary material for: The TLR7/9 adaptors TASL and TASL2 mediate IRF5-dependent antiviral responses and autoimmunity in mouse
Source: Nat Commun. 2025 Jan 24;16:967. doi: 10.1038/s41467-024-55692-y (PMC11759703; doi:10.1038/s41467-024-55692-y)
Supplement: Supplementary file 1 — Supplementary Information [file 41467_2024_55692_MOESM1_ESM.pdf]

Supplementary information

TLR7/9 adaptors TASL and TASL2 mediate IRF5-dependent antiviral responses and autoimmunity in mouse.

Ales Drobek, Léa Bernaleau, Maeva Delacrétaz, Sandra Calderon Copete, Claire Royer-Chardon, Mélissa Longepierre, Marta Monguió-Tortajada, Jakub Korzeniowski, Samuel Rotman, Julien Marquis, and Manuele Rebsamen*.

* **Corresponding Author:**
Manuele Rebsamen
Email: manuele.rebsamen@unil.ch

This file contains:

- Supplementary Figure 1-10
- Supplementary Table 1

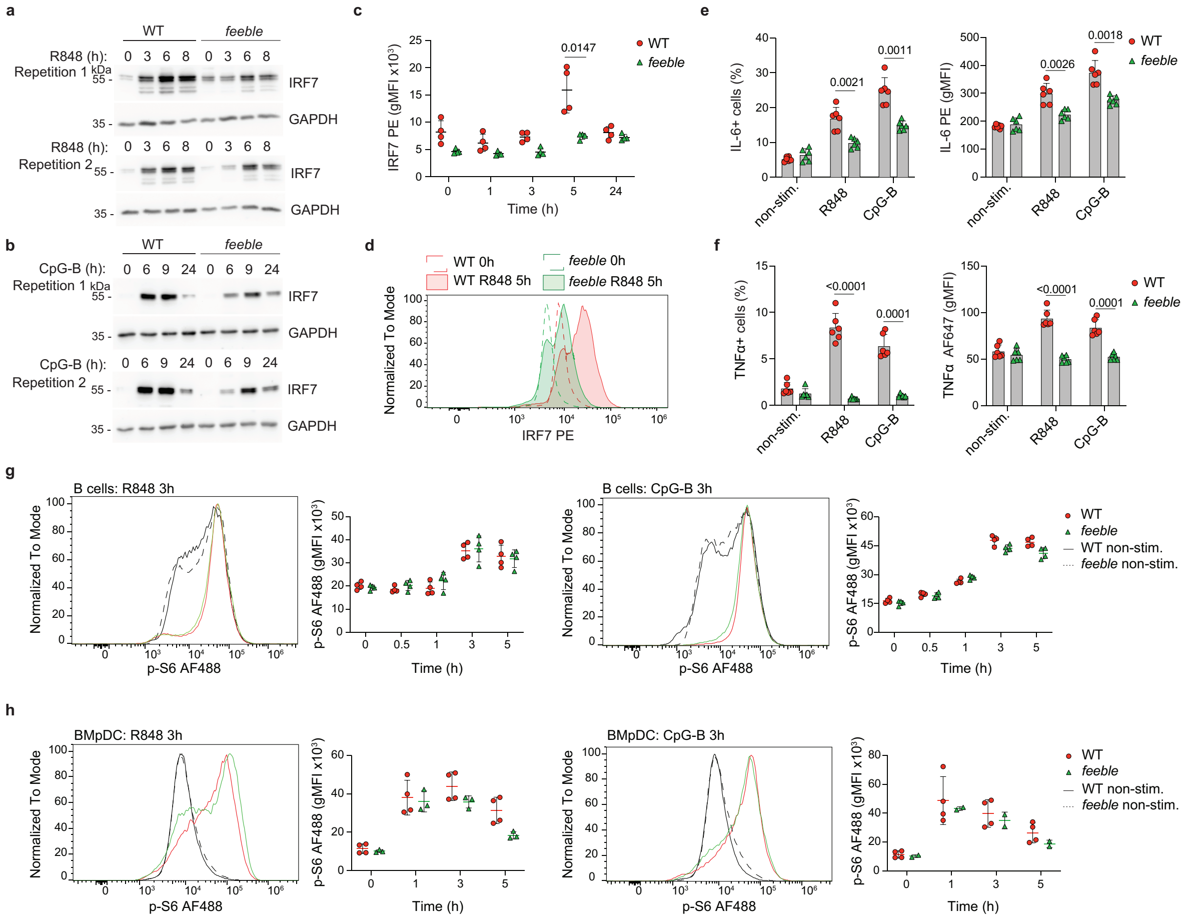

Supplementary Figure 1. mTORC1 activity is not significantly altered in *feeble* cells.

a-b, WT and *feeble* BM-pDC were stimulated with R848 (100 ng ml^{-1}) (a) or CpG-B (0.5 µM) (b) for the indicated time and lysates analyzed by immunoblotting for IRF7 induction. Repetition 1= males, Repetition 2= females in (a) and females in (b).

c-d, Quantification of IRF7 induction in BM-pDC after R848 (100 ng ml^{-1}) treatment measured by intracellular staining and quantified by gMFI over time (c), with representative histogram at 5h post activation (d). WT n=4 (2/2), *feeble* n=4 (2/2). Mean ± s.d..

e-f, Intracellular staining of splenic B cells for IL-6 (e) and TNFα (f) after o/n treatment (R848 100 ng ml^{-1}; CpG-B 0.5 µM) in the presence of Brefeldin A. Data represent % of positive and gMFI. WT n=6 (0/6), *feeble* n=6 (0/6). Mean ± s.d..

g-h, Intracellular staining of splenic B cells (g) or BM-pDC (h) for phospho-S6 at indicated timepoint after stimulation. Shown as representative histograms and quantified as gMFI. WT n=4 (4/0), *feeble* n=4 (2/0-2). Mean ± s.d.

Analysed by two-way ANOVA with Šidák's multiple comparisons test (c) or two-sided unpaired t test (e, f). n=total number (males/females).

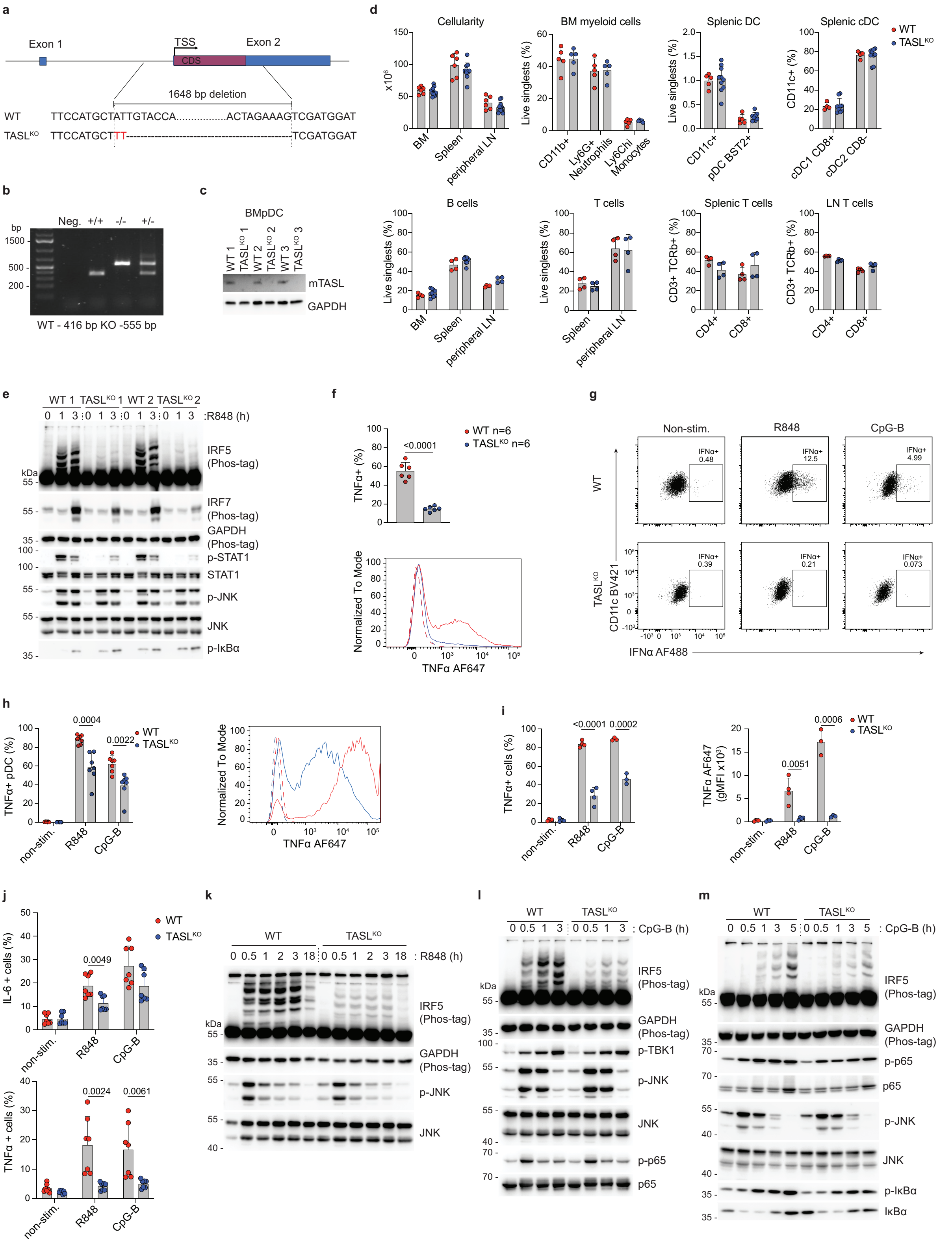

Supplementary Figure 2. TASL^{KO} shows partial defect in IRF5 activation.

a, Targeting strategy for deletion of *Tasl* coding region by the CRISPR/Cas9 and resulting deletion.

b, Genotyping PCR to distinguish between WT, KO and heterozygote.

c, Verification of TASL deletion on protein level by immunoblotting BM-pDC lysates from 3 independent WT and TASL^{KO} pairs (2 male and 1 female pairs).

d, Immunophenotyping of $TASL^{KO}$ compared to WT (7-8 weeks old males and females). B cells defined as $B220^+$ $CD19^+$, T cells gated as $CD3\varepsilon^+$ $TCR\beta^+$. WT n=4 (2/2), $TASL^{KO}$ n=4 (2/2). Mean ± s.d.

e, WT and $TASL^{KO}$ male BM-pDC were stimulated with R848 (5 μg ml^{-1}) and analyzed by immunoblotting.

f, Intracellular staining of TNFα in BM-pDC after o/n activation with R848 (5 μg ml^{-1}). Quantified by percentage of positive cells (top panel). Representative histogram is shown (bottom panel). WT n=6 (3/3), $TASL^{KO}$ n=6 (3/3). Mean ± s.d.

g, Representative dotplot of intracellular IFNα staining in splenic pDC after 3h stimulation (R848 100 ng ml^{-1}; CpG-B 0.5 μM).

h-i, Intracellular staining of TNFα in splenic pDC after 3h (h) or o/n (i) activation shown as percentage of positive cells, gMFI and representative histogram. WT n=7 (4/3), $TASL^{KO}$ n=7 (4/3) in (h) and WT n=4 (4/0), $TASL^{KO}$ n=4 (4/0) in (i). Mean ± s.d.

j, Intracellular staining for IL-6 (top) and TNFα (bottom) in splenic B cells after o/n activation (R848 5 μg ml^{-1}; CpG-B 5 μM). WT n=7 (7/0), $TASL^{KO}$ n=7 (7/0). Mean ± s.d.

k-l, WT and $TASL^{KO}$ splenic B cells were stimulated with R848 (5 μg ml^{-1}) (k) or CpG-B (5 μM) (l) and cells lysates analyzed by immunoblotting. Females (k) and males (l) were used as a source of primary cells.

m, WT and $TASL^{KO}$ female BM-pDC were stimulated with CpG-B (0.5 μM) and cells lysates analyzed by immunoblotting.

In (d, f, h, i, j) analysis performed with two-sided Mann-Whitney test. In (k-m) data are representative of at least two independent experiments. n=total number (males/females).

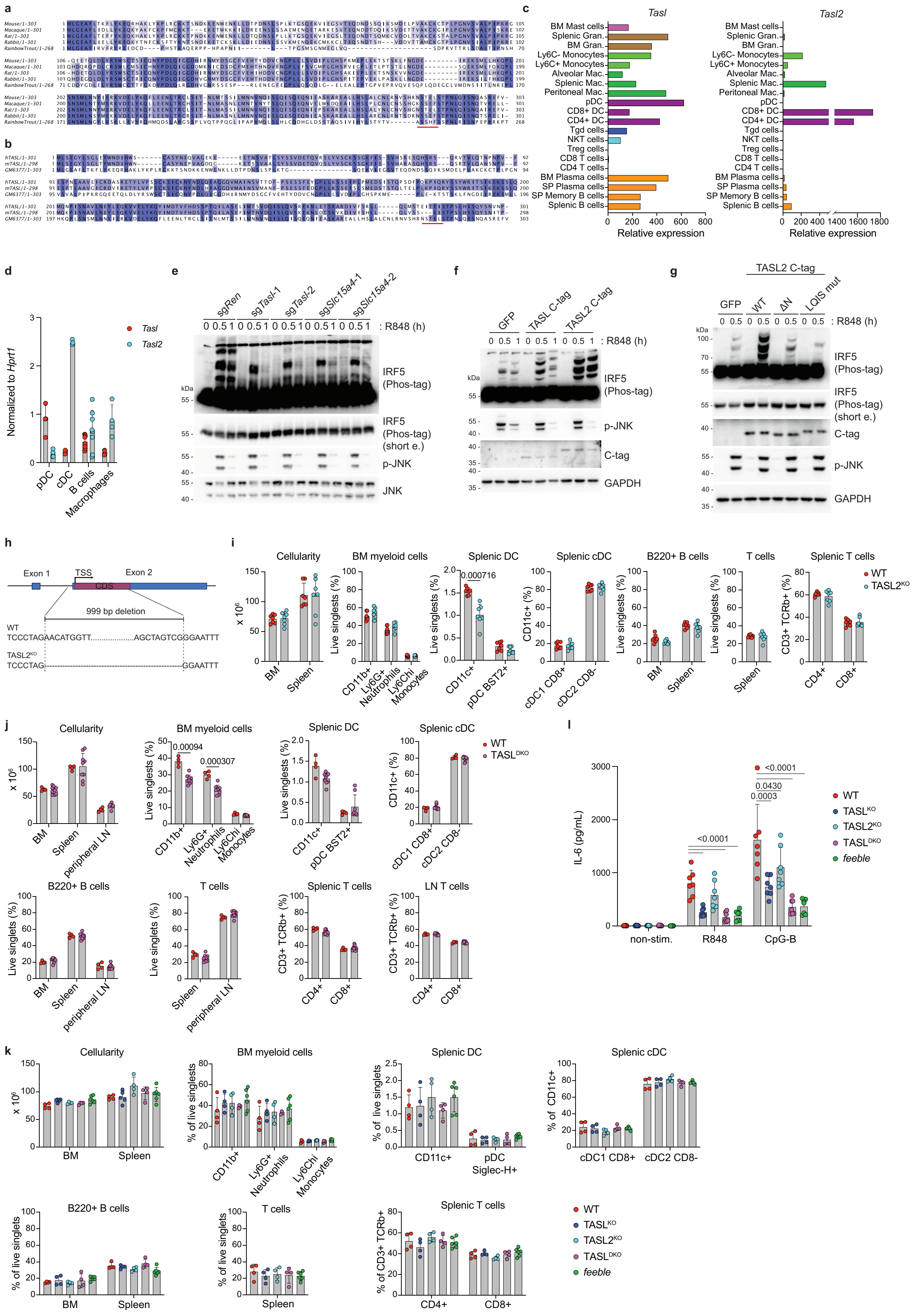

Supplementary Figure 3. TASL2 is a paralogue of TASL and can compensate for its function.

a, Protein sequence alignment of GM6377/TASL2 across species.
b, Protein sequence alignment of TASL (human and mouse) and TASL2 (GM6377).
c, Expression of *Tasl* and *Tasl2* in selected immune cell population (Source: ImmGen datasets, Expression Value Normalized by DESeq2).
d, *Tasl* and *Tasl2* expression in sorted splenic pDC (Siglec-H^{+} CD11c^{+}), cDC Siglec-H^{-} CD11c^{high}) or MACS enriched B cells and macrophage populations by RT-qPCR. 2-6 males and 2 females were used as a source of primary cells. Mean ± s.d.
e, IRF5 activation after R848 (5 μg ml^{-1}) treatment in control (sg*Ren*), *Tasl (*sg*Tasl)* or *Slc15a4 (*sg*Slc15A4)*-deleted RAW 264.7 cell lines.
f-g, IRF5 activation after R848 (5 μg ml^{-1}) treatment in RAW 264.7 cells stably overexpressing GFP, TASL or TASL2 (f), including TASL2 N-terminal and C-terminal deletion/mutation (g). ΔN: deletion of first 2-8 amino acids, LQIS mut → AQAA mutation.
h, Targeting strategy for deletion of TASL2 coding region on X chromosome by the CRISPR/Cas9 and resulting deletion.
i-j, Immunophenotyping of adult (8-12 weeks old) TASL2^{KO} (i) or TASL^{DKO} (j) mice. WT n=7 (3/4), TASL2^{KO} n=7 (3/4) in (i) and WT n=4 (4/0), TASL^{DKO} n=9 (5/4) in (j). Mean ± s.d.
k, Immunophenotyping of aged mice (6-8 months old) from the indicated genotypes. WT n=4 (2/2), TASL^{KO} n=4 (2/2), TASL2^{KO} n=4 (2/2), TASL^{DKO} n=4 (2/2), *feeble* n=4 (2/2). Mean ± s.d.
l, IL-6 production by splenic B cells across different genotypes after 24h stimulation *in vitro* (R848 100 ng ml^{-1}; CpG-B 0.5 μM). WT n=7 (3/4), TASL^{KO} n=7 (2/5), TASL2^{KO} n=7 (3/4), TASL^{DKO} n=7 (3/4), *feeble* n=7 (3/4). Mean ± s.d.
Two-sided Mann-Whitney test (i-j) or One-way ANOVA with Dunnet's multiple comparisons test compared to WT (k-l). In (e-g) data are representative of at least two independent experiments. n=total number (males/females).

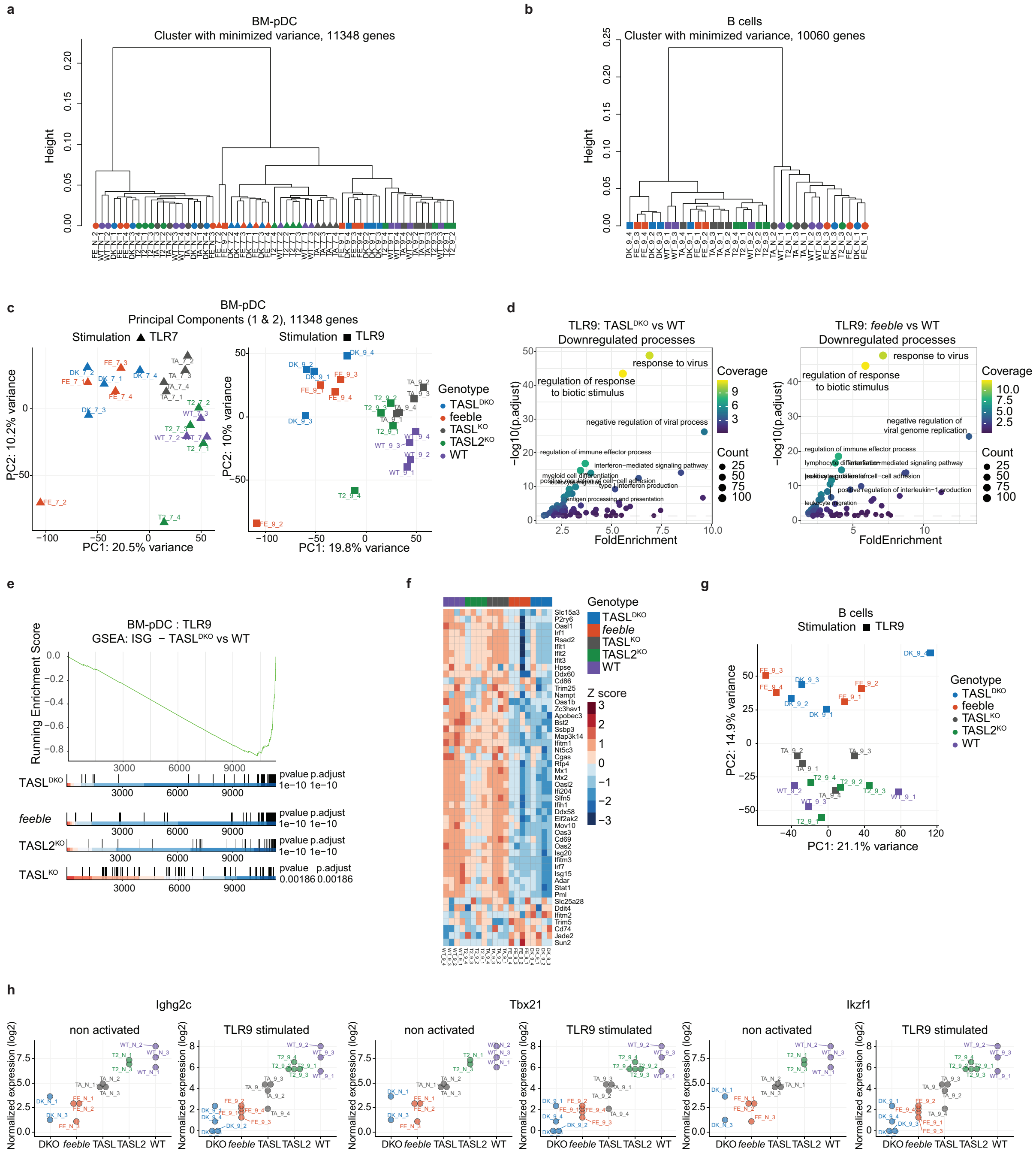

Supplementary Figure 4. Transcriptional responses in BM-pDC and B cells after *in vitro* TLR7/9 stimulation.

a-b, Hierarchical clustering of BM-pDC (a) or B cell (b) samples (non-stimulated and stimulated). TA=TASL^{KO}, T2=TASL2^{KO}, DK=TASL^{DKO}, FE=*feeble*, first number refers to activation (7 for TLR7 = R848 100 ng ml^{-1}; 9 for TLR9 = CpG-B 0.5 µM, N=non-stimulated), second number refers to biological replicate. Dot color corresponds to genotype and shape refers to activation status.

c, Principal component analysis of BM-pDC stimulated with R848 100 ng ml^{-1} (left panel) or CpG-B 0.5 µM (right panel).

d, Gene ontology (GO) analysis of downregulated processes in $TASL^{DKO}$ (left panel) and *feeble* (right panel) BM-pDC stimulated with CpG-B 0.5 μM, compared to WT. Each dot represents an enriched GO term (adjusted P < 0.05) with color indicating the coverage and gene count determining the size.

e, Gene set enrichment analysis of an ISG signature in BM-pDC from the indicated genotypes stimulated with CpG-B 0.5 μM compared to WT. Gene list were ranked by statistic. The upper panel depicts the running enrichment score specifically for $TASL^{DKO}$, and the lower panels show the ranking of ISG signature in each genotype.

f, Heatmap of ISG signature genes in TLR9 stimulated BM-pDC. Color intensity represents scaled expression levels.

g, Principal component analysis of TLR9 stimulated (CpG-B 0.5 μM) B cells.

h, Expression level of the indicated genes (*Ighg2c*, *Tbx21*, *Ikzf1*) in unstimulated and CpG-B stimulated B cells across all genotypes.

Female mice were used as a source of primary B cells and BM-pDC for transcriptional profiling.

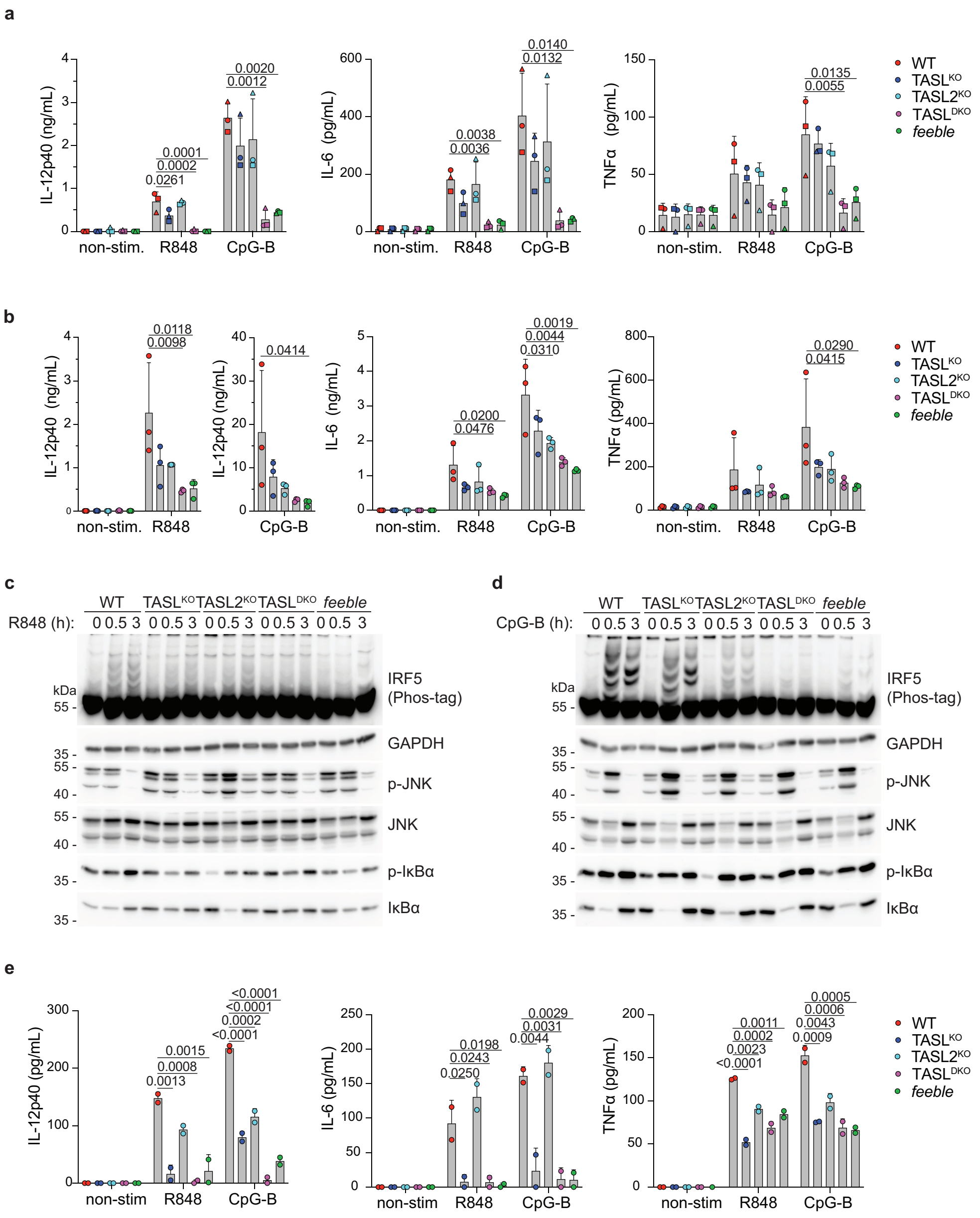

Supplementary Figure 5. Impaired responses in cDC and macrophages upon *ex vivo/in vitro* endosomal TLR agonist treatment.

a-b, Cytokines production in female (n=3) splenic cDC (a) or BM-cDC (b) after stimulation with R848 (100 ng ml^{-1}) or CpG-B (0.5 μM) for 24h. Mean ± s.d..

c-d, Male BM-pDC of indicated genotypes were treated with R848 (100 ng ml^{-1}) (c) or CpG-B (0.5 μM) (d) and analyzed by immunoblotting.

e, Cytokines production in female (n=2) splenic macrophages (F4/80^{+}) after stimulation with R848 (100 ng ml^{-1}) or CpG-B (0.5 μM) for 24h. Mean ± s.d..

In (c-d) data are representative of at least two independent experiments. Statistical analysis was performed with one-way ANOVA using Dunnet's multiple comparisons test compared to WT.

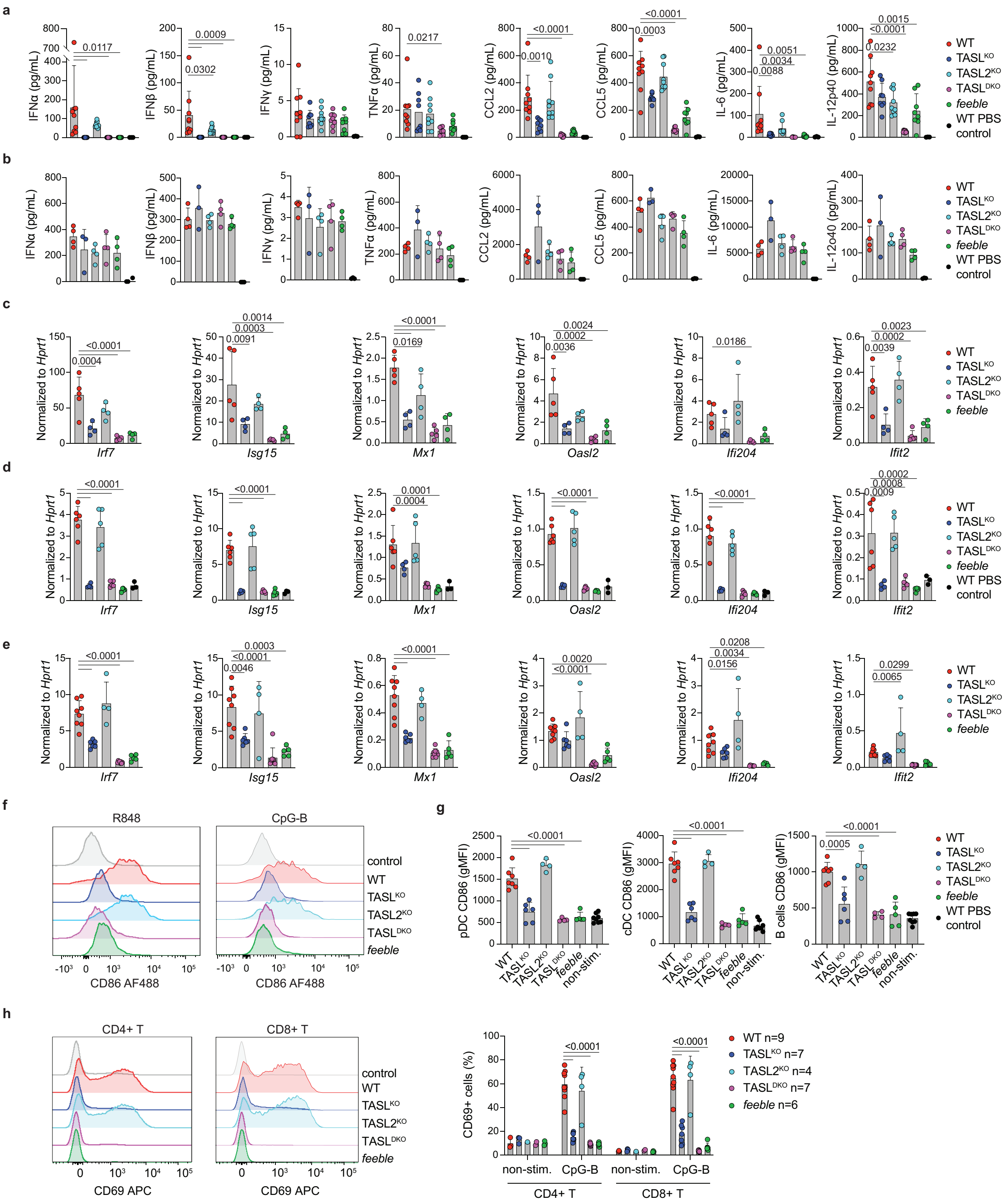

Supplementary Figure 6. Impaired type I IFN responses and cell activation upon *in vivo* endosomal TLR agonist challenge.

a-b, Type I IFN, cytokine and chemokine levels in serum of CpG-B injected (5 µg, 4h) (a) or LPS injected (20 µg, 2h) (b) mice. WT n=9 (4/5), TASL^{KO} n=8 (4/4), TASL2^{KO} n=9 (4/5), TASL^{DKO} n=8 (4/4), *feeble* n=8 (4/4) in (a) and WT n=5 (0/5), TASL^{KO} n=3 (0/3), TASL2^{KO} n=4 (0/4), TASL^{DKO} n=4 (0/4), *feeble* n=4 (0/4) in (b). Mean ± s.d..

c, Expression of the indicated ISGs in FACS-sorted splenic pDC 24h after CpG-B i.v. injection, analyzed by RT-qPCR. WT n=5 (3/2), TASL^{KO} n=4 (2/2), TASL2^{KO} n=4 (4/0), TASL^{DKO} n=5 (3/2), *feeble* n=4 (4/0). Mean ± s.d..

d-e, Expression of the indicated ISGs in FACS-sorted splenic cDC 24h after R848 (d) or CpG-B (e) i.v. injection, analyzed by RT-qPCR. WT n=6 (2/4), $TASL^{KO}$ n=4 (2/2), $TASL2^{KO}$ n=5 (2/3), $TASL^{DKO}$ n=5 (2/3), *feeble* n=5 (1/4) in (d) and WT n=8 (6/2), $TASL^{KO}$ n=6 (4/2), $TASL2^{KO}$ n=4 (4/0), $TASL^{DKO}$ n=8 (6/2), *feeble* n=5 (5/0) in (e). Mean ± s.d..
f, Representative histogram of CD86 upregulation on splenic pDC 24h after R848 (left) or CpG-B (right) i.v. injection.
g, gMFI quantification of CD86 upregulation on indicated splenic immune cell populations 24h after CpG-B injection measured by flow cytometry. WT n=7 (5/2), $TASL^{KO}$ n=6 (3/3), $TASL2^{KO}$ n=4 (4/0), $TASL^{DKO}$ n=4 (2/2), *feeble* n=5 (5/0). Mean ± s.d..
h, Upregulation of CD69 on splenic CD4 and CD8 T cells 24h after CpG-B treatment *in vivo*. Representative histogram (left) with quantification of $CD69^+$ T cells (right) are shown. WT n=9 (7/2), $TASL^{KO}$ n=7 (4/3), $TASL2^{KO}$ n=4 (4/0), $TASL^{DKO}$ n=7 (5/2), *feeble* n=6 (6/0). Mean ± s.d..
Statistical analysis was performed with one-way ANOVA using Dunnet's multiple comparisons test compared to WT. n=total number (males/females). WT controls represent either PBS injected control mice (b, d) or PBS+DOTAP injected control mice (a, c, e, g).

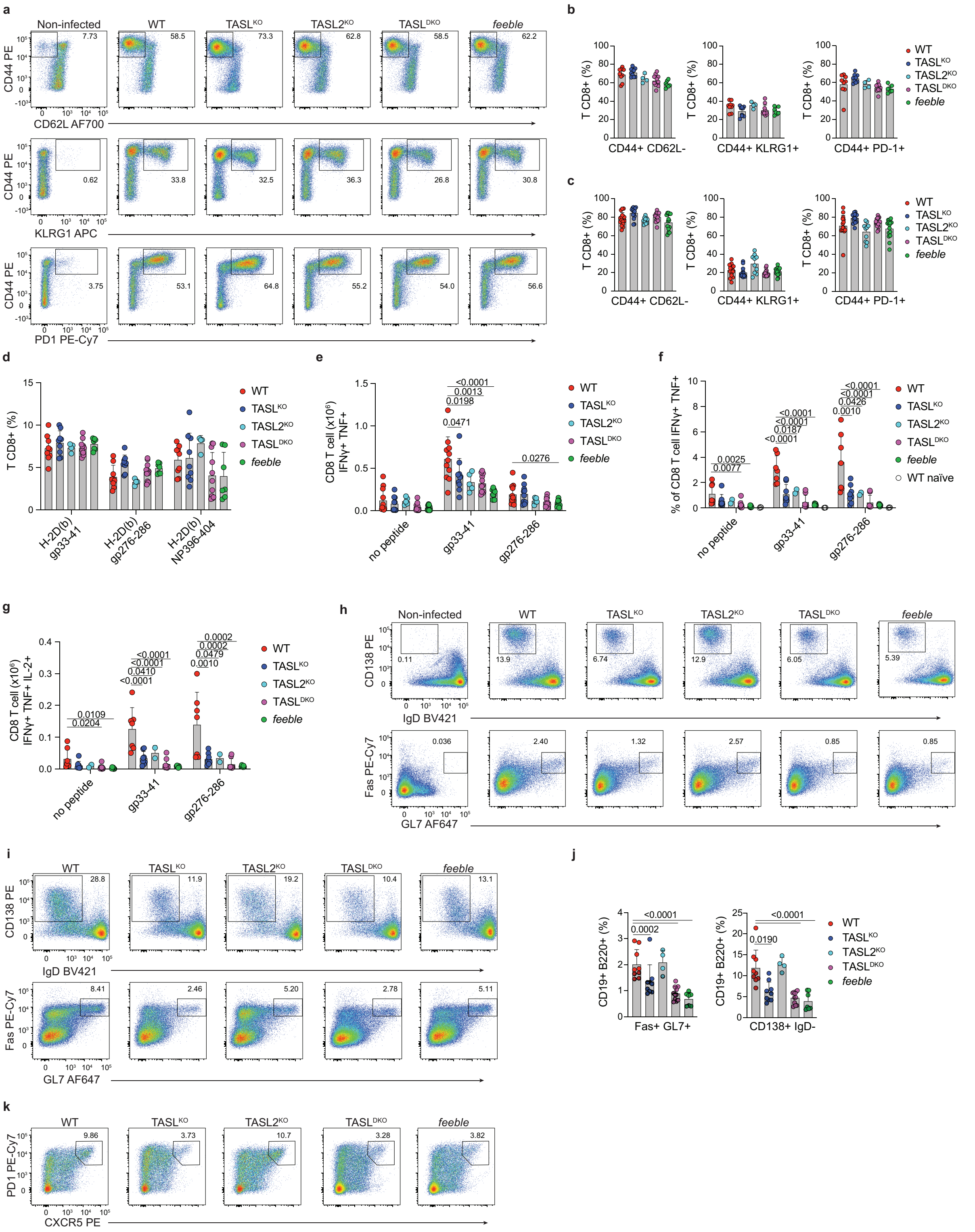

Supplementary Figure 7. Early T and B cell responses in LCMV infected mice.

a-c, Representative dot-plot of $CD8^+$ T cell response in spleen 8 days after infection (a). Formation of effector cells (CD44+ $CD62L^-$), cytotoxic ($CD44^+$ $KLRG1^+$) and $PD1^+$ cells in all genotypes is shown (a). These populations were quantified on day 8 (b) and day 15 (c) p.i. WT n=9 (6/3), $TASL^{KO}$ n=9 (5/4), $TASL2^{KO}$ n=4 (0/4), $TASL^{DKO}$ n=11 (6/5), *feeble* n=7 (4/3) in (b) and WT n=16 (4/12), $TASL^{KO}$ n=15 (4/11), $TASL2^{KO}$ n=10 (2/8), $TASL^{DKO}$ n=16 (6/10), *feeble* n=15 (2/13) in (c). Mean ± s.d..

d, MHC class I tetramer positive splenic CD8 T cells were analyzed on day 8 p.i.. WT n=9 (6/3), $TASL^{KO}$ n=9 (5/4), $TASL2^{KO}$ n=4 (0/4), $TASL^{DKO}$ n=11 (6/5), *feeble* n=7 (4/3). Mean ± s.d..

e-g, Frequency or absolute number of double (e-f) or triple (g) cytokine producing splenic $CD8^+$ T cells from 8 days (e) or 3 months (f-g) infected mice restimulated *in vitro* with indicated peptides for 5h and analyzed by flow cytometry. WT n=11 (6/5), $TASL^{KO}$ n=10 (5/5), $TASL2^{KO}$ n=5 (0/5), $TASL^{DKO}$ n=11 (6/5), *feeble* n=9 (4/5) in (e) and WT n=7 (7/0), $TASL^{KO}$ n=8 (8/0), $TASL2^{KO}$ n=2 (2/0), $TASL^{DKO}$ n=8 (8/0), *feeble* n=7 (7/0) in (f-g). Mean ± s.d..

h-j, Representative dot-plot of short-lived plasma cells (IgD^- $CD138^+$) and germinal center B cells ($GL7^+$ Fas^+) formation in pre-gated splenic B cell population ($B220^+$/low $CD19^+$/low) at day 8 p.i. (h) with their quantification (j). WT n=9(6/3), $TASL^{KO}$ n=9(5/4), $TASL2^{KO}$ n=4(0/4), $TASL^{DKO}$ n=11(6/5), *feeble* n=7(4/3). Mean ± s.d.. Representative dot-plot of short-lived plasma cells and GC B cells at day 15 p.i. (i).

k, Representative dot-plot of Tfh cells formation in CD4+ T cells 15 days p.i.

Analysis performed with one-way ANOVA with Dunnet's multiple comparisons test compared to WT. n=total number (males/females). WT naïve represent non-infected control mice.

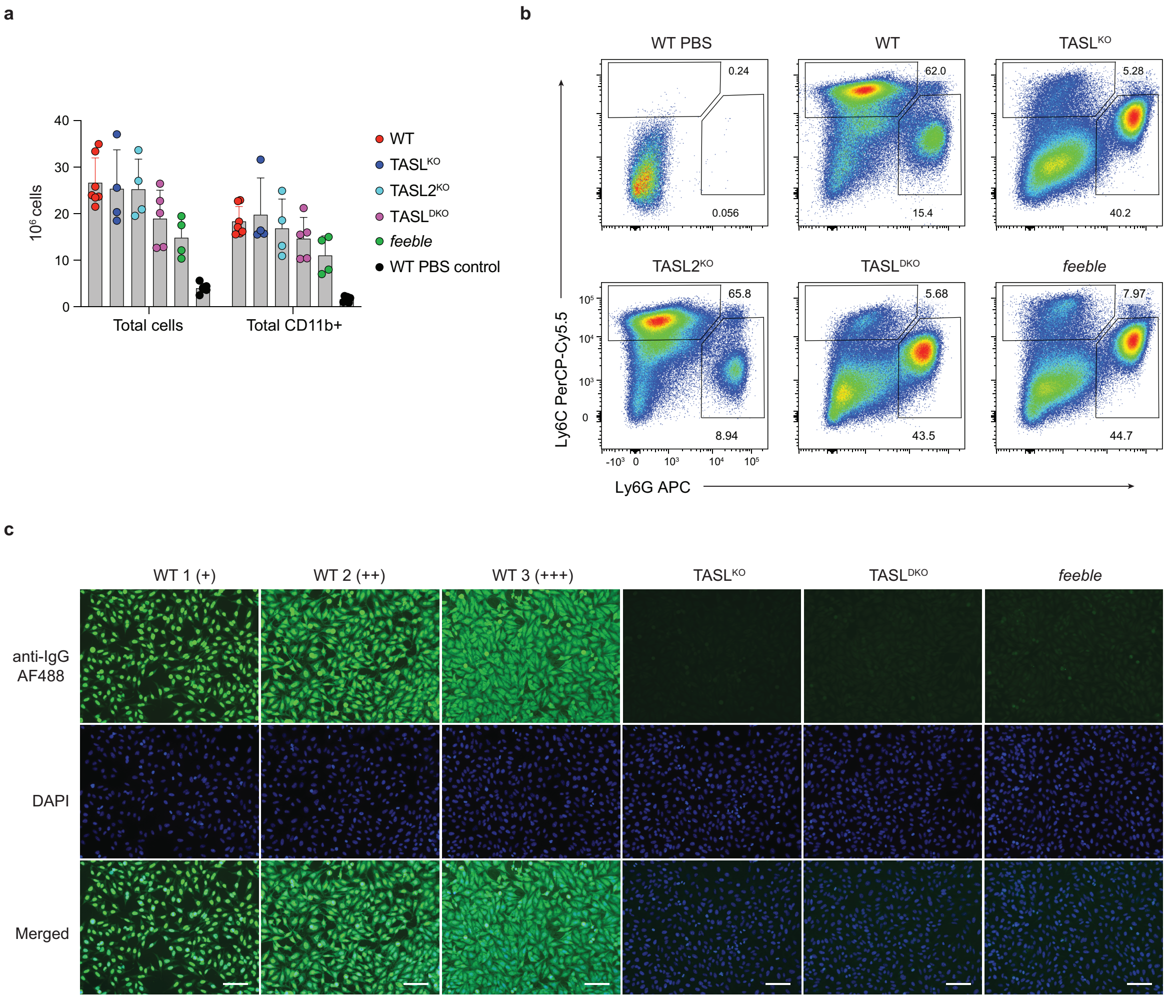

Supplementary Figure 8. Peritoneal immune cell infiltration after pristane treatment.

a, Count of total cells and myeloid cells (CD11b^{+}) infiltrating peritoneum 14 days after pristane injection measured by flow cytometry. WT PBS control represent PBS injected mice. WT n=7 (7/0), TASL^{KO} n=4 (4/0), TASL2^{KO} n=4 (4/0), TASL^{DKO} n=5 (3/2), *feeble* n=4 (4/0). Mean ± s.d..

b, Representative dot-plot of pre-gated myeloid (CD11b^{+}) cell populations showing proinflammatory monocytes (Ly6C^{high}) and neutrophils (Ly6G^{+}) in peritoneum 14 days post injection as quantified in Fig. 7a. WT PBS represents PBS injected control mouse.

c, Antinuclear antibodies (ANA) in serum 6 months after pristane treatment, assessed by Hep-2 immunofluorescence. Representative images for indicated genotypes showing different pattern and intensity of staining (+ to +++) in WT samples. Scale = 100 µm.

n=total number (males/females).

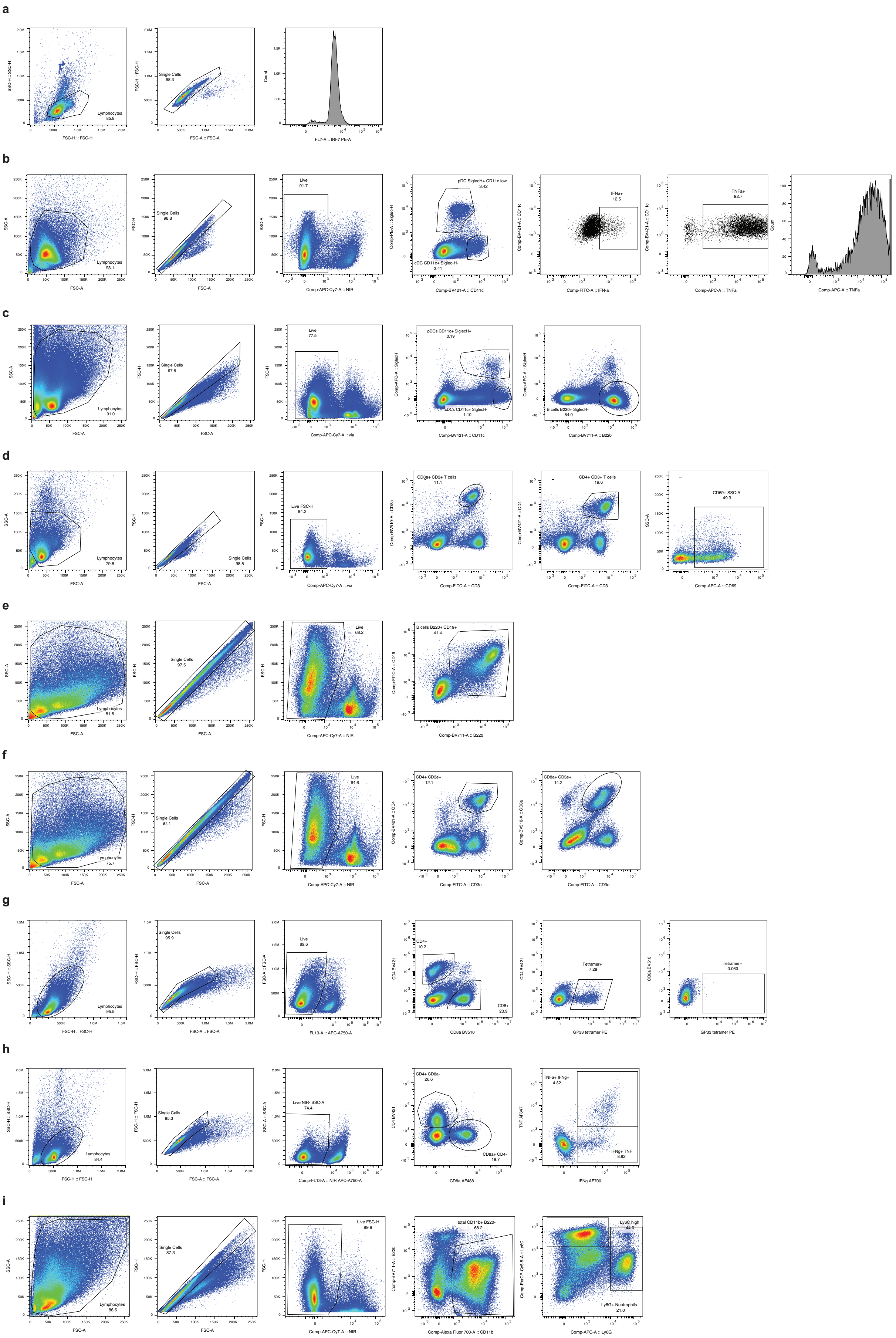

Supplementary Figure 9. Gating strategy for the flow cytometry analysis.

a, Gating strategy used for fixed cell flow cytometry analysis for experiments in Fig. 1c and Supplementary Fig. 1g-h.
b, Gating strategy used for fixed cell flow cytometry analysis for experiments in Fig. 2d-e and Supplementary Fig. 2g-h.
c, Gating strategy used for live cell flow cytometry analysis for experiments in Fig. 5c and Supplementary Fig. 6g.
d, Gating strategy used for live cell flow cytometry analysis for experiments in Supplementary Fig. 6h.
e, Gating strategy used for live cell flow cytometry analysis for experiments in Fig. 6f and Supplementary Fig. 7h, i, j.
f, Gating strategy used for live cell flow cytometry analysis for experiments in Fig. 6g and Supplementary Fig. 7a, b, c, k.
g, Gating strategy used for live cell flow cytometry analysis for experiments in Supplementary Fig. 7d.
h, Gating strategy used for fixed cell flow cytometry analysis for experiments in Fig. 6d, e and Supplementary Fig. 7e, f, g.
i, Gating strategy used for live cell flow cytometry analysis for experiments in Fig. 7a and Supplementary Fig. 8a, b.

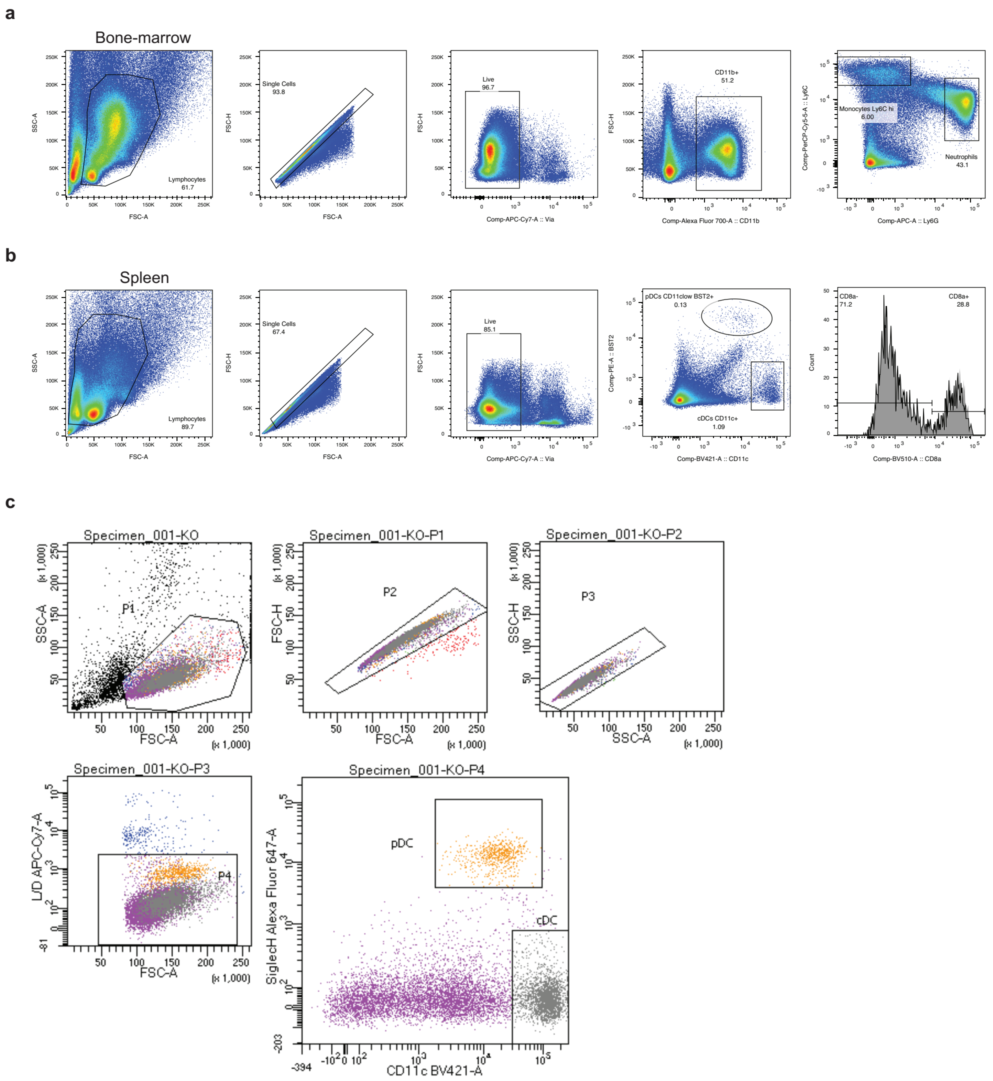

Supplementary Figure 10. Gating strategy for the flow cytometry analysis of steady state immune system (immunophenotyping) and cell sorting.

a-b, Gating strategy used for live cell flow cytometry analysis of bone-marrow (a) and spleen (b) for experiments in Supplementary Fig. 2d, 3i, j, k.

c, Gating strategy used for fluorescence activated cell sorting of splenic pDC and/or cDC for experiments in Fig. 2f, g, 5b and Supplementary Fig. 3d, 5a, 6c, d, e.

qPCR		
**Name**	**Forward**	**Reverse**
mIFNa	CTCCTAGACTCATTCTGCAATG	CAGGCACAGGGGCTGTGTT
mIFNb1	CCCTATGGAGATGACGGAGA	CTGTCTGCTGGTGGAGTTCA
mTNF	CACCACGCTCTTCTGTCTAC	AGGGTCTGGGCCATAGAACT
mIL-6	GATGGATGCTACCAAACTGGAT	TGAAGGACTCTGGCTTTGTCT
mIl12b	ATGGAGTCATAGGCTCTGGAAA	CCGGAGTAATTTGGTGCTTCAC
mHPRT1	GCAAACTTTGCTTTCCCTGGT	CTGGCCTGTATCCAACACTTC
mISG15	ACCTAGAGCTAGAGCCTGCA	CCAATCTTCTGGGCAATCTGC
mMx1	GTTGACTACCACTGAGATGACC	CAGGATGAAGTACTGGATAATCAG
mOasl2	TGCCTGCTGCAGGTCTGTTG	TCCAGAGTGTCCAATCCACTG
mIfi204	GGACATTTGTGAGTGGAGAGTAC	CTGCCTGGTTCACACCTGAC
mIRF7	CTGAAGTGAGGGGGGTCCAG	CACAGCCCAGGCCTTGAAGA
LCMV 13	ACAACTCCCACCATTACATCAG	CTATACTCATGAGTGTGTGGTC
**LCMV specific cDNA synthesis**		
LCMV cDNA2	CTTGGGAAAGGAGAAT	
**Genotyping**		
Tasl_genot_common	TGCTGTCTAGACCCCAGGAA	
Tasl_genot_KOrev		AGCACTTGTGGGATAGAAGCA
Tasl_genot_WTrev		ACAGTGGTAGGCACAAAGGT
Gm6377 common	GTAAATTCTTCAGGGTTAAACAGTG	
Gm6377 WTrev		GAAAGTGAATTGTCCGGTGTATC
Gm6377 KOrev		TCCCAGGGTATTCTATTTGTCAGTG
mSLC15A4	GAAGCTACAACATACATAAGAGCC	TTAGGTTAAAGATCGAGGTCCAG
**sgRNA – cell lines**		
sgRen	CACCGGTATAATACACCGCGCTAC	AAACGTAGCGCGGTGTATTATACC
sgSlc15a4 No.1	CACCGGGCGCGCCGTTCGACTGGGA	AAACTCCCAGTCGAACGGCGCGCCC
sgSlc15a4 No.2	CACCGCTCACCTGATCGGCGCCGAA	AAACTTCGGCGCCGATCAGGTGAGC
sgTasl No.1	CACCGTGCATCTTATAATGAACCG	AAACCGGTTCATTATAAGATGCAC
sgTasl No.2	CACCGCAACGACTTACATATTGCAG	AAACCTGCAATATGTAAGTCGTTGC
**sgRNA – mice**		
Tasl sgRNA 1	ATGGGGCTGGTACAATAGCA	
Tasl sgRNA 2	ATGGCAAACTAGAAAGTCGA	
Gm6377 sgRNA 1	AACAGTTCTTCCCTAGAACA	
Gm6377 sgRNA 2	AAATATCAAACCAAGCTAGT	

Supplementary Table 1. List of oligonucleotides used in this study.
