# Supplementary material for: The TLR7/9 adaptors TASL and TASL2 mediate IRF5-dependent antiviral responses and autoimmunity in mouse
Source: Nat Commun. 2025 Jan 24;16:967. doi: 10.1038/s41467-024-55692-y (PMC11759703; doi:10.1038/s41467-024-55692-y)
Supplement: Supplementary file 2 — Description of Additional Supplementary Files [file 41467_2024_55692_MOESM2_ESM.pdf]

Description of Additional Supplementary Files

File Name: Supplementary Data 1
Description: RNA-seq (related to Fig.4 and Supplementary Fig.4). Normalized number of reads (DESeq2) for all BM-pDC samples.

File Name: Supplementary Data 2
Description: RNA-seq (related to Fig.4 and Supplementary Fig.4). Normalized number of reads (DESeq2) for all splenic B cell samples.

File Name: Supplementary Data 3
Description: Simplified list of GOBP (Gene Ontology Biological Process) related to Fig. 4c for TLR7 activated BM-pDC - $TASL^{DKO}$ vs WT (Downregulated processes).

File Name: Supplementary Data 4
Description: Simplified list of GOBP (Gene Ontology Biological Process) related to Fig. 4c for TLR7 activated BM-pDC - *feeble* vs WT (Downregulated processes).

File Name: Supplementary Data 5
Description: Simplified list of GOBP (Gene Ontology Biological Process) related to Supplementary Fig. 4d for TLR9 activated BM-pDC - $TASL^{DKO}$ vs WT (Downregulated processes).

File Name: Supplementary Data 6
Description: Simplified list of GOBP (Gene Ontology Biological Process) related to Supplementary Fig. 4d for TLR9 activated BM-pDC - *feeble* vs WT (Downregulated processes).
