# Supplementary material for: The TLR7/9 adaptors TASL and TASL2 mediate IRF5-dependent antiviral responses and autoimmunity in mouse
Source: Nat Commun. 2025 Jan 24;16:967. doi: 10.1038/s41467-024-55692-y (PMC11759703; doi:10.1038/s41467-024-55692-y)
Supplement: Supplementary file 9 — Reporting Summary [file 41467_2024_55692_MOESM9_ESM.pdf]

nature portfolio

Corresponding author(s): Rebsamen

Last updated by author(s): Nov 6, 2024

Reporting Summary

Nature Portfolio wishes to improve the reproducibility of the work that we publish. This form provides structure for consistency and transparency in reporting. For further information on Nature Portfolio policies, see our Editorial Policies and the Editorial Policy Checklist.

Statistics

For all statistical analyses, confirm that the following items are present in the figure legend, table legend, main text, or Methods section.

n/a	Confirmed	
☐	☒	The exact sample size (*n*) for each experimental group/condition, given as a discrete number and unit of measurement
☐	☒	A statement on whether measurements were taken from distinct samples or whether the same sample was measured repeatedly
☐	☒	The statistical test(s) used AND whether they are one- or two-sided<br>*Only common tests should be described solely by name; describe more complex techniques in the Methods section.*
☒	☐	A description of all covariates tested
☐	☒	A description of any assumptions or corrections, such as tests of normality and adjustment for multiple comparisons
☐	☒	A full description of the statistical parameters including central tendency (e.g. means) or other basic estimates (e.g. regression coefficient) AND variation (e.g. standard deviation) or associated estimates of uncertainty (e.g. confidence intervals)
☐	☒	For null hypothesis testing, the test statistic (e.g. *F*, *t*, *r*) with confidence intervals, effect sizes, degrees of freedom and *P* value noted<br>*Give P values as exact values whenever suitable.*
☒	☐	For Bayesian analysis, information on the choice of priors and Markov chain Monte Carlo settings
☒	☐	For hierarchical and complex designs, identification of the appropriate level for tests and full reporting of outcomes
☒	☐	Estimates of effect sizes (e.g. Cohen's *d*, Pearson's *r*), indicating how they were calculated

Our web collection on statistics for biologists contains articles on many of the points above.

Software and code

Policy information about availability of computer code

Data collection	Flow cytometry and FACS sorting data were collected using BD FACSDiva or BeckmanCoulter CytExpert software. Images were acquired by Zeiss AxioVision software (SE64 Rel.4.9.1). ELISA readouts were recorded by SoftMax Pro (v7.1) or Multiscan FC (v1.01.16). RT-qPCR data were collected by LightCycler 480 SW 1.5.1. Immunoblot images were acquires by Fusion software Evolution-Capt Edge (v18-02). Luminex (ProcartaPlex) data were acquired by Bio-Plex Data Pro software 1.2.
Data analysis	Flow cytometry data were analyzed using BD FlowJo v10.9. Flow cytometry, ELISA, ProcartaPlex, viral load and RT-qPCR data were analyzed using Microsoft Excel and Graphpad Prism v9. Image analysis was performed using ImageJ2 (v2.14.0). BRB-seq data were analyzed by R software package.

For manuscripts utilizing custom algorithms or software that are central to the research but not yet described in published literature, software must be made available to editors and reviewers. We strongly encourage code deposition in a community repository (e.g. GitHub). See the Nature Portfolio guidelines for submitting code & software for further information.

Data

Policy information about availability of data

All manuscripts must include a data availability statement. This statement should provide the following information, where applicable:

- Accession codes, unique identifiers, or web links for publicly available datasets
- A description of any restrictions on data availability
- For clinical datasets or third party data, please ensure that the statement adheres to our policy

Data supporting the findings of this study are available within the paper, its Supplementary Information, and source data file. Primary BRB-seq data are deposited and available under GEO accession number (GSE267786).

Research involving human participants, their data, or biological material

Policy information about studies with human participants or human data. See also policy information about sex, gender (identity/presentation), and sexual orientation and race, ethnicity and racism.

Reporting on sex and gender	N/A
Reporting on race, ethnicity, or other socially relevant groupings	N/A
Population characteristics	N/A
Recruitment	N/A
Ethics oversight	N/A

Note that full information on the approval of the study protocol must also be provided in the manuscript.

Field-specific reporting

Please select the one below that is the best fit for your research. If you are not sure, read the appropriate sections before making your selection.

☒ Life sciences ☐ Behavioural & social sciences ☐ Ecological, evolutionary & environmental sciences

For a reference copy of the document with all sections, see nature.com/documents/nr-reporting-summary-flat.pdf

Life sciences study design

All studies must disclose on these points even when the disclosure is negative.

Sample size	No statistical method was used to predetermine sample size.
Data exclusions	No data were excluded from the study except several BRB-seq sample replicates if they didn't reach minimal number of reads for analysis or exhibiting outlier behavior as described in Methods section.
Replication	Replication of experiments performed within this study is explicitly stated in Figure legends either as number of biological replicates (n=x) and/or number of independent replications.
Randomization	No randomisation was performed as mice were allocated to experimental groups based on the genotype.
Blinding	The Investigators were not blinded to experimental mice allocation as this was determined by the genotype. Blinding is not relevant for the experiments performed as the results are quantitative.

Reporting for specific materials, systems and methods

We require information from authors about some types of materials, experimental systems and methods used in many studies. Here, indicate whether each material, system or method listed is relevant to your study. If you are not sure if a list item applies to your research, read the appropriate section before selecting a response.

Materials & experimental systems

n/a	Involved in the study
	[x] Antibodies
	[x] Eukaryotic cell lines
[x]	Palaeontology and archaeology
	[x] Animals and other organisms
[x]	Clinical data
[x]	Dual use research of concern
[x]	Plants

Methods

n/a	Involved in the study
[x]	ChIP-seq
	[x] Flow cytometry
[x]	MRI-based neuroimaging

Antibodies

Antibodies used

For flow cytometry analysis, directly labeled antibodies from Biolegend were used: CD11b Alexa Fluor 700 (1012229, Lot:B323579, dilution 1/200), CD11c Brilliant Violet 421 (117343, Lot:B318328, B347721, 1/200), Siglec-H AF647 (129608, Lot:B271031, 1/200), BST2 PE (127010, Lot:B344506, 1/200), F4/80 Alexa Fluor 488 (123120, Lot:B272102, 1/200), Ly6C PerCP-Cy5.5 (128011, Lot:B282010, 500x), Ly6G Alexa Fluor 647 (127610, Lot:B255839, 1/200), MHCII (I-Ab) PE-Cy7 (116419, Lot:B289598, 1/400), CD8a Brilliant Violet 510 (100752, Lot:B336696, 1/200), B220 Brilliant Violet 711 (103255, Lot:B326247, B391673, 1/200), CD19 FITC (115521, Lot:B306929, 1/200), IgM Brilliant Violet 421 (405725, Lot:B293444, 1/200), IgD Alexa Fluor 700 (405729, Lot:B255981, 1/200), CXCR4 APC (146507, Lot:B278597, 1/200), CD138 PE (142503, Lot:B322376, 1/200), CD3e Alexa Fluor 488 (100321, Lot:B289380, 1/200), CD4 Brilliant Violet 421 (100437; Lot:B297643, B357846, 1/200), TCR-beta Brilliant Violet 785 (109249, Lot:B336011, 1/200), NK1.1 APC (108709, Lot:B316224, 1/200), CD44 PE (103007, Lot:B295248, 1/600), CD62L Alexa Fluor 700 (104426, Lot:B333915, 1/200), CD25 PE-Cy7 (102015, Lot:B290258, 1/200), KLRG1 APC (138412, Lot:B344824, 1/200), CD279 (PD1) PE-Cy7 (135215, Lot:B355883, 1/200), CD185 (CXCR5) BV605 (145513, Lot:B324823, 1/200), CD185 (CXCR5) PE (145503, Lot:B350818, 1/200), CD95 (Fas) PE-Cy7 (152617, Lot:B351840, 1/200), GL7 Alexa Fluor 647 (144606, Lot:B351840, 1/200), CD86 AF647 (105019, Lot:B308606, 1/200), CD86 AF488 (105018, Lot:B364517, 1/200), CD69 APC (104513, Lot:B361543, 1/200), TNFa AF647 (506314, Lot:B263283, 1/200 or 1/500 overnight), IL-6 (504503, Lot:B353935, 1/200), TCR-beta biotin (109204, Lot:B279070, 1/500), B220 biotin (103204, Lot:B335405, 1/500), Ly6G biotin (127604, Lot:B314607, 1/500), CD19 biotin (115504, Lot:B313073, 1/500), Isotype control IgG1, kappa, PE (RTK2071, Lot:B398003, 1/100), Isotype control IgG2a, kappa, Alexa Fluor 647 (RTK2758, Lot:B407447, 1/200).

From ThermoFisher: C-tag biotin (7103252100, Lot:181002-01, 1/1000).

Invitrogen: Goat anti-Rabbit AF488 (A11034, Lot:2286890, 1/1000), goat anti-Rabbit Alexa Fluor 568 (A-11011, 1/1000), Podocin (PA5-79757, Lot:ZC4251691, 1/500), IRF-7 PE (12-5829-82, Lot:2410125, 1/100), phospho-IRF5 Ser437 (PA5-106093, Lot:YK4113295B, 1/1000)

PBL assay science: Anti-mouse interferon Alpha (32100-1, Lot:7558, 1/50).

Antibodies against V5 (80076S), HA (3724), SAPK/JNK (9252), phospho-SAPK/JNK Thr183/Tyr185 (4668), NF-κB p65 (8242), phospho-NF-κB p65 Ser536 (3033), IκBα (4814), phospho-IκBα Ser32 (2859), STAT1 (14994), phospho-STAT1 Tyr701 (7649), phospho-TBK1/NAK Ser172 (5483) and IRF7 (72073) were from Cell Signaling and all used at 1/1000 dilution.

IRF5 (ab181553, Lot:GR3248905-4, GR3278824-6, 1/1000) was from Abcam

GAPDH (sc-365062, Lot:I2321, 1/1000) was from Santa Cruz.

Biotinylated antibodies used in cells purification F4/80 biotin (130-116-514, Lot: 5221100468, 1/200), NK1 biotin (130-120-513, Lot:5221100457, 1/200), Siglec-H biotin (130-101-858, Lot:5220909807, 1/100) were from Miltenyi.

SigmaAldrich (Merck): Cxorf21 (HPA001185, Lot:000030856, 1/500).

Conjugated antibodies anti-mouse IgG HRP (115-035-003, Lot:161655, 1/5000), anti-rabbit IgG HRP (111-035-003, Lot:161658, 1/5000) used as secondary antibodies in Western Blot, goat anti-rat HRP (112-035-062, 1/2500) used in LCMV focus forming assay and anti-mouse IgG AF488 (115-545-166, Lot:167698, 1/1000).

BioXCell: Rat anti-LCMV (BE0106, Lot:787521S1, 1/500)

Custom rabbit antibody against GM6377 was generated with GenScript and used at 1/500 dilution.

Validation

Antibodies were bought from commercial vendors and validation for indicated species and applications can be found on the manufacturers website or the provided scientific citations on the same website by entering the catalog number of respective manufacturer:
https://www.biolegend.com/
https://www.cellsignal.com/
https://www.jacksonimmuno.com/
https://www.thermofisher.com/
https://www.miltenyibiotec.com/

https://www.sigmaaldrich.com/
https://www.abcam.com/
https://www.scbt.com/
https://bioxcell.com/

Custom made antibody against GM6377 (TASL2) by Genscript was validated on HEK293T transfected cells with Gm6377 coding plasmid. This antibody does not detect endogenous level of GM6377. Antibody against TASL (Cxorf21, HPA001185, SigmaAldrich) was validated on western blot using set of WT and TASL-KO Bone marrow derived plasmacytoid dendritic cells (BM-pDC) samples.

Eukaryotic cell lines

Policy information about cell lines and Sex and Gender in Research

Cell line source(s)	HEK293T (CRL-3216) and RAW 264.7 (TIB-71) were purchased from ATCC. Flt3-L producing cells (B16 melanoma) were a kind gift from Prof. Steven Porcelli (Albert Einstein College of Medicine, NY, USA). Primary cells were isolated from age and sex matched animals.
Authentication	Cell lines were obtained directly from ATCC and were not validated further.
Mycoplasma contamination	All cell lines were negative for mycoplasma as revealed by regular PCR testing and MycoStrip (Invivogen) testing.
Commonly misidentified lines (See ICLAC register)	None of commonly misidentified cell lines were used in this study.

Animals and other research organisms

Policy information about studies involving animals; ARRIVE guidelines recommended for reporting animal research, and Sex and Gender in Research

Laboratory animals	The mice used in experiments had C57BL/6J background (Charles River). Experimental animals were 6-12 weeks old at the time/start of experiments. We aimed at constant inclusion of males and females in experiments. The used transgenic strains were: TASL and GM6377 (TASL2) knockouts generated with CRISPR-Cas9 by Centre for Transgenic Models - CTM, Basel, Switzerland . Slc15a4-deficient feeble mice (034296-JAX; C57BL/6J-Slc15a4m1Btlr/Mmjax) were obtained from Jackson Laboratory.
Wild animals	The study did not include wild animals.
Reporting on sex	Both male and female mice were used for experiments. Animals were age and sex matched for each experiment and the number of animals and their sex is described in figure legends for each panel. Sex of individual data points is clearly indicated in Source data file for each figure panel.
Field-collected samples	The study did not include field collected samples.
Ethics oversight	All experiments involving animals were performed under the guidelines of and with approval from the cantonal veterinary office of the canton of Vaud (Switzerland), license number VD3716 and VD3779.

Note that full information on the approval of the study protocol must also be provided in the manuscript.

Plants

Seed stocks	N/A
Novel plant genotypes	N/A
Authentication	N/A

Flow Cytometry

Plots

Confirm that:

- [x] The axis labels state the marker and fluorochrome used (e.g. CD4-FITC).
- [x] The axis scales are clearly visible. Include numbers along axes only for bottom left plot of group (a 'group' is an analysis of identical markers).
- [x] All plots are contour plots with outliers or pseudocolor plots.
- [x] A numerical value for number of cells or percentage (with statistics) is provided.

Methodology

Sample preparation	Single cell suspensions from the spleen, lymph nodes, and bone marrow were prepared by gentle meshing the organs with syringe plungers or flushing open bones with FACS buffer (PBS, 2% FBS. 2 mM EDTA). Cells were harvested by centrifugation at 400g at 4° C for 5 min. Pellets were resuspended in 1 ml ACK buffer for the red blood cell lysis for 2-4min, diluted with FACS buffer, pelleted again, resuspended and processed for flow cytometry analysis.
Instrument	BD LSRII, BeckmanCoulter Cytoflex S, BD FACSAria III
Software	FlowJo 10.9.0 (BD Biosciences), FACSDiva, CytExpert software
Cell population abundance	Splenic pDC cells were sorted from magnetically pre-enriched spleen (depletion of CD19+ and TCRbeta+ cells). Abundance of pDCs before sort ranged between 2-10 %. Because of low number of sorted cells, post-sort re-analysis was not performed. Based on percentage of pDCs and total number of cells in the samples, the sorting efficiency was approximately 70 %.
Gating strategy	The lymphocytes were gated using FSC vs SSC. The singlets were gated based on the area vs height of FSC. Viable cells were gated based on the exclusion of LIVE/DEAD Near-IR viability dye (ThermoFisher) positive cells. In the following steps, cell subsets were gated based on the antibody used as shown in the Supplementary Figures.

- [x] Tick this box to confirm that a figure exemplifying the gating strategy is provided in the Supplementary Information.
